# 

**Published:** 1896-01

**Authors:** 


### SELECTIONS.

Article I.—Pyorrhea Alveolaris. By Dr. B. F. Arrington, - 385
" II.—Dr. Paine's Local Anesthetic. - - - - - 399
" III.—Systemic Medication for the Relief of Sensitive Dentine, - - - - - - - 403
" IV.—Dr. L. P. Haskell, on Dental Prosthesis, - - 406
" V.—From German Dental Journals. By Carl E. Klotz, L. D. S., - - - - - - - 415
" VI.—Died in a Dentist's Chair, - - - - - 418
" VII.—Caries of Jaw From Impacted Wisdom Tooth. By R. E. Sparks, - - - - - - 420

### OBITUARY.

Dr. James E. Garretson, - - - - - - - - 422

### EDITORIAL, ETC.

To the Members of the Maryland State Dental Association and the Washington City Dental Society, - - - - - 426
Strange Case of a Degenerating Human Body, - - - 429
Dentist's Gold, - - - - - - - - - 430

### MONTHLY SUMMARY.

Borine, - - - - - - - - - - 430

Men That Should not be Tolerated in the Dental Profession. - 431

Filling Children's Teeth. - - - - - - - - 431

Varnishing Cavities. - - - - - - - 432

Pulp-Capping in Deciduous Teeth, - - - - - - 432

